# Is There a Link Between Autistic People Being Perceived Unfavorably and Having a Mind That Is Difficult to Read?

**DOI:** 10.1007/s10803-019-04101-1

**Published:** 2019-06-13

**Authors:** Rabi Samil Alkhaldi, Elizabeth Sheppard, Peter Mitchell

**Affiliations:** University of Nottingham, University Park, Nottingham, NG7 2RD UK

**Keywords:** Autism, First impressions, Mind reading, Social interaction, Person perception

## Abstract

The link between autistic people having a mind that is difficult to read (by neurotypical participants) and being perceived unfavorably was investigated. Videoed Autistic and neurotypical targets from Sheppard et al. (PLOS ONE 7(11):e49859, 2016) were scored for how readable they were when reacting to a distinctive greeting from the experimenter. These videos were presented to new groups of perceivers (neurotypical adults) who rated neurotypical targets more socially favorably than autistic targets irrespective of whether details of the experimenter’s greeting were concealed (Study 1) or disclosed (Study 2). Target readability correlated with ratings of target favorability (r = .58 and r = .63), independent of target diagnosis. Perceivers might rate targets unfavorably because they experience difficulty reading them, though other interpretations of the correlation are also possible.

Much research has investigated how effectively autistic people read others’ minds, and some results suggest poor ability and developmental delay in this arena (Baron-Cohen 1995). Having difficulty reading others’ minds is likely to be socially disadvantageous, for one might struggle to sense how others are feeling, what they are intending to do and what they are trying to communicate, amongst other things. But it could also be socially disadvantageous to have a mind that is difficult for others to read. Milton (2012) theorised that whenever interactants hold differing social norms and expectations (as is often the case for autistic and non-autistic individuals), there is a tendency for a ‘disjuncture in reciprocity’, or lack of empathy. He referred to this as a ‘double empathy problem’, where difficulties in social interaction experienced by autistic individuals result from bidirectional failures of autistic and non-autistic interaction partners to read each other’s minds. Recent evidence suggests autistic people have minds that are difficult for others (perceivers) to read, including autistic perceivers (Edey et al. 2016) as well as neurotypical perceivers (Sheppard et al. 2016). Among neurotypical participants, having a mind that is difficult to read is connected with being perceived unfavorably (Anders et al. 2016), which could place one at risk of social exclusion with associated consequences for poor mental health (Mitchell 2017). Autistic individuals do indeed tend to be perceived unfavorably by non-autistic others (Sasson et al. 2017) and also have elevated risk of mental health problems compared with the rest of the population (Cassidy and Rodgers 2017). Bringing all these pieces of circumstantial information together raises the possibility of an association between autistic people being perceived unfavorably by non-autistic people and being difficult to read. The purpose of this article is to report the first empirical investigation into this possibility.

Two recent studies report that people (perceivers) find it difficult to read autistic individuals (targets). The most recent (Edey et al. 2016) used a variant of the classic task devised by Heider and Simmel (1944) with targets invited to manipulate geometrical shapes to enact interpersonal emotions like coaxing, mocking, seducing and surprising. While doing so, the movement of the shapes was video recorded. These recordings were later shown to perceivers tasked with inferring which interpersonal emotion was being enacted on any given occasion. Perceivers (irrespective of whether they were autistic) were more accurate in their inferences when the targets (those generating the movements of the geometric shapes) were neurotypical than autistic. The authors of the study suggested this is a sign that the actions (and therefore the minds) of autistic people are relatively difficult to interpret or read.

A study by Sheppard et al. (2016) offers converging evidence from a task that employed naturalistic stimuli. Targets were greeted by the experimenter on arrival either with a compliment, a joke, a story relating the experimenter’s difficult day or the experimenter neglecting the target while making a personal telephone call (following a procedure developed by Pillai et al. 2012). Unknowingly, the targets were video recorded during the greeting scenario and another group of non-autistic participants who viewed the target videos (perceivers) were tasked with inferring which particular greeting each target was experiencing. Notably, but unknown to perceivers, half the targets were autistic and half were neurotypical. Overall, perceivers were considerably more accurate in inferring the greeting scenario when viewing a typically developing target than when viewing an autistic target (except for the ‘joke’ scenario); effectively, perceiver accuracy judgments discriminated between autistic and non-autistic targets.

This task is defined as ‘retrodictive mindreading’ (Gallese and Goldman 1998; Teoh et al. 2017), where perceivers make an inference of the target’s inner state from the signal in the target’s observable behavior. They then make a further inference from the target’s behavior/inferred inner state to the event that caused the target to experience such a state. The perceiver thus makes a proximal inference (the target’s inner state) and a distal inference (the event that precipitated the target’s inner state). This is how we operationally define mindreading in the current research and in doing so we are not making any assumptions about how perceivers fare in inferring what the target believes as being true of the world and inferring how the target feels (see Poletti et al. 2012, for a review of the complexity of this issue).

Apparently, then, the signal to the target’s inner state is not as clear when the target is autistic than when they are not autistic. One might wonder if this is because autistic targets are less expressive than non-autistic targets. Some evidence is consistent with such a possibility (Macdonald et al. 1989; Stagg et al. 2014) but a considerable amount of evidence suggests autistic people are at least as expressive as non-autistic people in many/most contexts (Beadle-Brown and Whiten 2004; Press et al. 2010; Volker et al. 2009), though the form of expressiveness can be rather different in autism (Faso et al. 2015). Sheppard et al. (2016) sought clarity by asking an independent group of perceivers to rate the videos specifically on the intensity of target expressions. Generally, autistic targets were not less expressive than non-autistic targets and there was no basis for suggesting perceivers were inaccurate in inferring what autistic targets were reacting to *because* autistic targets were inexpressive. Rather, it seems the signal emitted by autistic targets, even though expressive, was more prone to being misinterpreted compared with the signal emitted by non-autistic targets. In short, autistic people are difficult for non-autistic people to read.

Autistic people also tend to be perceived unfavorably by other people. Sasson et al. (2017) video recorded targets while engaging in a short social interaction and these videos were subsequently presented to non-autistic perceivers who had no prebriefing alerting them to the possibility that some targets were autistic. Perceivers rated the targets on a series of scales relating to social favorability and their ratings effectively discriminated between autistic and non-autistic targets: The former were perceived less socially favorable. A follow-up study supported this basic finding but the results also suggested that disclosing the diagnostic status of targets to perceivers had a discounting effect, such that perceiver ratings became considerably more positive for autistic targets (Sasson and Morrison 2017).

Anders et al. (2016) examined the relationship between readability and likeability (which presumably has something in common with social favorability) in a study involving neurotypical targets. In the study, perceivers were tasked with inferring whether targets were expressing fear or sadness and their accuracy in this respect correlated with various direct and indirect ratings on whether they liked (were attracted to) the targets. Although target expressions were posed rather than natural, tempering any conclusions about mindreading, the findings nevertheless offer circumstantial evidence to suggest a connection between target readability and likeability.

In summary, the evidence suggests autistic people are less readable than non-autistic people (Edey et al. 2016; Sheppard et al. 2016), that autistic people are perceived less favorably than non-autistic people (Sasson et al. 2017) and that when perceivers find it difficult to read targets, they also tend to perceive those targets as unlikeable (Anders et al. 2016). This evidence leads us to question whether there is an association between autistic people being difficult to read and autistic people being perceived unfavorably by non-autistic others. The purpose of the work presented here was to carry out the first empirical investigation into this possibility.

We re-analysed perceiver ratings archived from Sheppard et al. (2016) to calculate a readability score for each target who served in that study. We then presented these same target videos to a newly recruited group of non-autistic perceivers (in the absence of any briefing that some targets were autistic) who were asked to rate social favorability of each target, using an adaptation of the scale devised by Sasson et al. (2017). Our focal analysis compared target ratings of readability with target ratings of favorability to test the prediction of a positive correlation.

## Study 1

### Methods

#### Stimuli

The videos used in the study were developed by Sheppard et al. (2016). Target participants were filmed within a testing room as they reacted naturally to the researcher’s behavior in one of four scenarios (joke, waiting, compliment and story), determined at random.

#### Participants (Targets)

The 40 targets, aged between 13 and 21 (M = 15.4 years) are described in Sheppard et al. (2016). Briefly, they were recruited from educational establishments and were all native speakers of English. Twenty were autistic, as evaluated by mental health professionals according to DSM-IV criteria (American Psychiatric Association 2013). All targets were undertaking basic or advanced level secondary or tertiary education courses. All were male Caucasians and the two groups were matched for chronological age.

#### Procedure

As described in Sheppard et al. (2016), a Sony DCR-SR60 video camera was placed about 1.7 m directly opposite the target, across the table on a tripod. As each target arrived individually at the testing room, the researcher performed (determined randomly) one of four possible scenarios (compliment, joke, story, waiting) while, the camera surreptitiously recorded the targets’ reactions. In the compliment scenario, the researcher paid the target a series of three compliments. In the joke scenario, the researcher told the target a joke. In the story scenario, the researcher told the target of several mishaps that had occurred earlier that day. In the waiting scenario, the researcher kept the target waiting while doing irrelevant activities such as text messaging. Five targets from each group experienced each scenario. In total, there were 40 edited video clips (one for each target with five target clips per scenario for each group) with a mean duration of 7.22 s. The video clips were 1080 pixels in width and 720 pixels in height, presented at 25 frames per second without sound (see Pillai et al. 2012, 2014; Sheppard et al. 2016 for details).

### Perceiver Phase

#### Participants (Perceivers)

Thirty-one typically developing perceivers (10 males and 21 females) aged between 20 and 28 (*M* = 24.5 years, *SD* = 1.98) were recruited through the ‘participant recruitment system’ and advertisements at the University of Nottingham. Although these perceivers were older than the targets on average (as with Sheppard et al. 2016), we assumed their data would still be informative about the association between target readability and favorability at least on a general level.

#### Procedures

The procedure for the study was approved by the School of Psychology Ethics committee, University of Nottingham (Ethics approval Number: S964). All 40 target videos were shown to each perceiver on a 15 in. MacBook Air, presented in random order using PsychoPy2 version 1.85.2 (Peirce 2007). Each perceiver (tested individually) viewed each video once only, and after viewing each they rated the target on nine social favorability dimensions on a scale from 1 to 6. Perceivers saw the target video first, then they saw a further screen with all nine questions (these were: willingness to talk to, awkwardness, attractiveness, intelligence, likeability, trustworthiness, dominance/submissiveness, self-esteem and empathy) which appeared in fixed order to avoid confusion from one trial to the next. Perceivers gave their rating to each question on a scale from 1 to 6, and rated questions from 1 to 9 in sequence:

How much would you like to talk to this person? How awkward is this person? How attractive is this person? How trustworthy is this person? How dominant is this person? How likable is this person? How intelligent is this person? How good is this person’s self-esteem? How empathic is this person?

Questions were adapted from Sasson et al. (2017). Due to possible redundancy, we excluded three of the questions used in the previous research (desire to live near the target, likelihood of hanging out with the target in their free time, level of comfort sitting next to the target), as they correlated highly with ‘likelihood of starting a conversation with the target’ (which was retained), ranging from .78 to .98. These exclusions gave an opportunity to add two further questions: ‘How good is this person’s self-esteem?’ and ‘How empathic is this person?’ No indication was given to the perceivers prior to the task that any of the individuals in the videos were autistic. Following completion of the task participants were debriefed and fully informed of the true purpose of the study.

### Results and Discussion

**Fig. 1:**
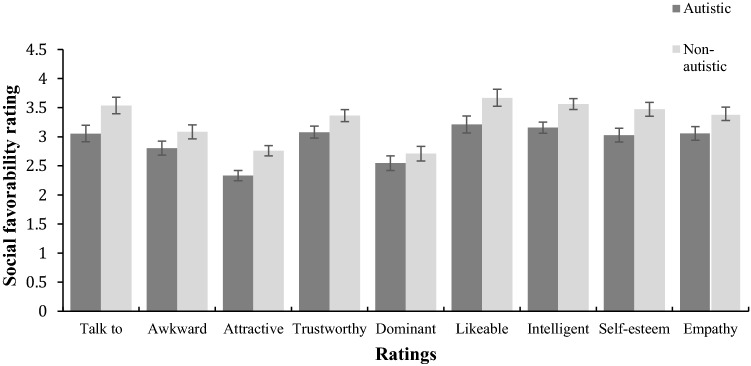
Ratings (1–6) by perceivers on nine dimensions of social favorability for autistic and non-autistic targets. Scoring for ‘awkwardness’ was reversed, such that a high rating was consistent with social favorability. The error bars represent the standard error of the mean

Perceivers rated each target on nine social favorability scales. We averaged ratings across all perceivers for each of these nine scales and with each target group. Mean ratings appear in Fig. 1.

Bivariate Pearson correlations revealed a strong positive correlation among all nine social favorability factors (Table 1). ‘Awkwardness’ correlated negatively with all other items (*r *> − 0.75, *p* < .001). A global ‘social favorability’ variable was derived from the sum of the averages of all nine scales (the reciprocal of the awkwardness item was used). This was justified given the high correlations between all measures, supported by the very high Cronbach’s alpha of .98, indicating strong internal reliability of the nine scales. A 2 × 4 repeated measures ANOVA was conducted to assess the associations between group and scenario type on global social favorability. A main effect of target group was found, *F*(1,30) = 104.14, *p *< .001, with autistic targets viewed less socially favorable than non-autistic targets. A main effect of scenario was also found, Greenhouse–Geisser corrected due to violation of sphericity, *F*(1.42, 42.65) = 86.57, *p *< .001. Post-hoc pairwise comparisons were conducted between the four scenario types, with Bonferroni correction: the main effect of scenario was driven by targets in the waiting scenario being rated significantly less socially favorable than those in all other scenarios (*p *< .001); there were no differences between the other three scenarios in how socially favorable targets were rated.

**Table 1:** Inter-correlations between nine scales of social favorability, all significant at *p* < .001 (2-tailed)

Variable	1	2	3	4	5	6	7	8	9
1. Talk to	–	− .89	.79	.94	.69	.97	.86	.91	.96
2. Awkward	–	–	− .76	− .83	− .79	− .86	− .75	− .91	− .87
3. Attractive	–	–	–	.70	.70	.76	.74	.80	.75
4. Trustworthy	–	–	–	–	.58	.96	.87	.83	.97
5. Dominant	–	–	–	–	–	.64	.60	.85	.62
6. Likeable	–	–	–	–	–	–	.85	.88	.96
7. Intelligent	–	–	–	–	–	–	–	.80	.85
8. Self-esteem	–	–	–	–	–	–	–	–	.86
9. Empathy	–	–	–	–	–	–	–	–	–

**Fig. 2:**
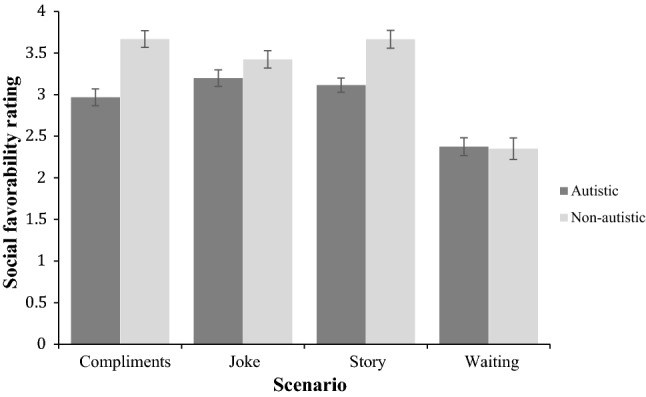
Perceivers’ ratings of the social favorability of autistic and non-autistic targets who experienced one of the four scenarios. The error bars represent one standard error of the mean

A significant interaction effect (Fig. 2) between group and scenarios was also found, *F*(3,90) = 29.65, *p *< .001, raising the possibility that perceiver ratings were more positive for one target group over the other, depending on the scenario. To explore this possibility, four post hoc paired-samples *t* tests were employed to compare participant performance across the two target groups for each scenario independently. Autistic targets were perceived less socially favorable than typically developing targets in the compliment scenario *t*(30) = 9.64, *p *< .001, Joke scenario *t*(30) = 4.08, *p *< .001, and story scenario *t*(30) = 8.11, *p *< .001. However, there was no significant difference in how socially favorable target groups were rated in the waiting scenario, t(30) = 0.44, *p *= .66, where both groups were rated rather negatively. Reported *p*-values are following Bonferroni correction. Summing up, it is notable that perceiver ratings of social favorability effectively discriminated between autistic and typically developing targets, even though perceivers were not given any information that the targets formed two discrete groups according to clinical diagnosis.

**Fig. 3:**
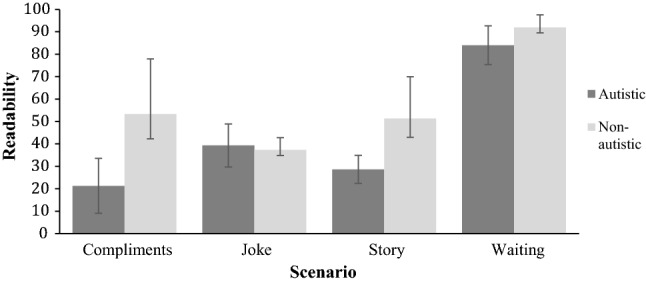
Perceivers’ readability of autistic and non-autistic targets. Each target participated in one of four scenarios. The error bars represent one standard error of the mean

Were autistic targets less readable than non-autistic targets? We conducted a confirmatory analysis by repurposing archived readability data from Sheppard et al. (2016) and calculating the number of times each target was judged as responding to the correct scenario. This was converted to a percentage to adduce a mean readability value associated with each target. A 2 × 4 repeated measures ANOVA was conducted on target readability, with the factors target group and greeting scenario. A main effect of target group was found, *F*(1,32) = 6.56, *p *= .015, confirming that autistic targets were less readable than non-autistic targets. A main effect of scenario was also found, *F*(3,32) = 17.467, *p *< .001, but the interaction term was nonsignificant, *F*(3,32) = 1.631, *p *= .202. Bonferroni corrected pairwise comparisons revealed that readability in the waiting scenario was significantly greater than in the other scenarios, as illustrated in Fig. 3.

**Fig. 4:**
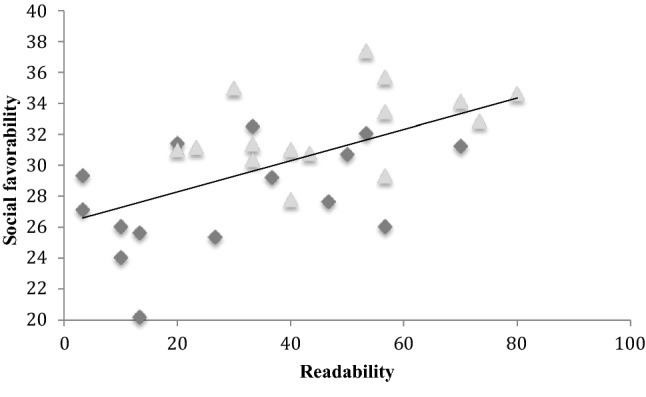
The correlation between readability and social favorability across autistic and non-autistic targets (waiting scenario excluded). The diamonds represent autistic targets and triangles represent non-autistic targets

Was the tendency for autistic targets to be perceived less positively associated with their being less readable? To begin to answer this question we conducted a bivariate correlation between target readability and target social favorability across three scenarios (excluding the waiting scenario). The waiting scenario was excluded because this condition yielded high readability perhaps because targets were merely waiting while the experimenter was apparently preoccupied with something else, whereas in the other scenarios the experimenter was actively engaging with the target. Hence, perceivers might easily identify this scenario through a process of elimination. Also, favorability ratings were quite different (much more negative) than for the other scenarios, irrespective of diagnosis, perhaps because targets appeared disagreeable on being neglected by the experimenter. Based on the remaining three scenarios (i.e. 30 pairs of values), we found a positive relationship between target social favorability and target readability, *r* = 0.58, *p *< .001, see Fig. 4. When participant group (autistic or non-autistic) was entered as a controlling variable in a partial correlation, the significant relationship between social favorability and readability survived, *r* = 0.45, *p *< .01. It seems the relationship between target social favorability and target readability is not a simple function of autistic targets being unreadable and also being perceived as rather less socially favorable. Perhaps the relation between readability and social favorability is more fundamental in a way that transcends target clinical status.

## Study 2

The second study offers a replication but with disclosure to perceivers of the particulars of the scenario each target was experiencing. Such information might help perceivers to contextualize target behavior, which should reduce noise in mean target favorability data. Accordingly, we might expect the correlation between target readability and favorability to be even stronger than in the first study. We might also find targets in the waiting scenario are no longer rated less favorably than in other scenarios when perceivers can interpret apparently disagreeable target behavior arising as a product of a disagreeable situation.

### Methods

#### Participants

Thirty perceivers (3 males and 27 females) aged between 18 and 25 (*M* = 19.8 years, *SD *= 1.94) took part; none had participated in Study 1. All were native English speakers.

#### Procedure

Each perceiver viewed the 40 target videos under the same conditions as in the first study and gave ratings according to the same nine questions (social favorability). In this study perceivers were informed which scenario each target was experiencing, and the name of each scenario appeared before each target video for 5 s.

### Results and Discussion

**Fig. 5:**
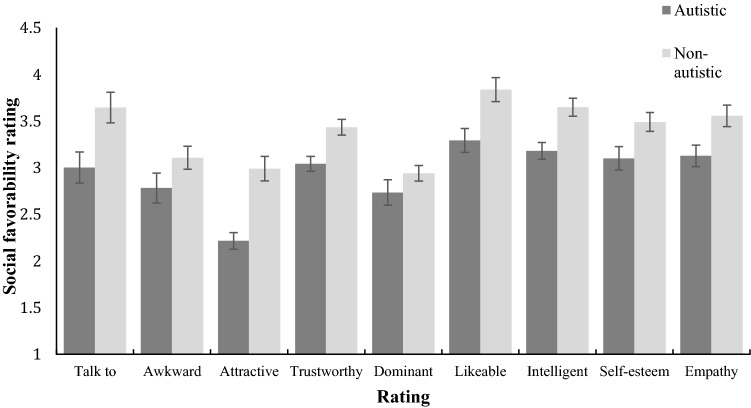
Ratings (1–6) by perceivers on nine dimensions of social favorability for autistic and non-autistic targets. Scoring for ‘awkwardness’ was reversed, such that a high rating was consistent with social favorability. The error bars represent the standard error of the mean

Group differences across the nine ratings of social favorability are apparent in Fig. 5. As with Study 1, a single social favorability variable was created from the ratings and submitted to a two-way repeated measures ANOVA to assess the effects of group and scenario type. A main effect of target group resulted from autistic targets being rated less favorably than typically developing targets, *F*(1,29) = 127.94, *p *< .001. A main effect of scenario was also found, F(2.11, 61.2) = 66.80, *p *< .001, Greenhouse–Geisser corrected for violation of sphericity. Post-hoc pairwise comparisons with Bonferroni correction revealed that targets in the waiting scenario were rated significantly less socially favorable than targets in other scenarios (*p *< .001); there were no differences between the other three scenarios in how socially favorable targets were rated.

**Fig. 6:**
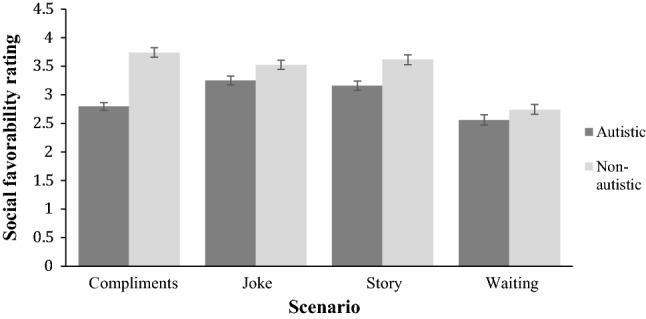
Perceivers’ ratings of social favorability of autistic and non-autistic targets who experienced one of the four scenarios. The error bars represent one standard error of the mean

A significant interaction effect (Fig. 6) between group and scenarios was also found, *F*(3,78.67) = 29.1, *p *< .001, raising the possibility that ratings were more positive for one target group than the other, depending on the scenario. To explore this possibility, four post hoc paired-samples *t* tests were employed to compare perceiver ratings across the two target groups for each scenario independently. Autistic targets were rated less socially favorable for the compliment scenario *t*(29) = − 10.26, *p *< .001, Joke scenario *t*(29) = − 4.75, *p *< .001, story scenario *t*(29) = − 6.60, *p *< .001 and waiting scenario, *t*(29) = − 3.98, *p *< .001. Reported *p*-values are following Bonferroni correction. To further explore the interaction, repeated measures ANOVAs were conducted to determine whether there was an effect of scenario for each group. For the autistic group, a main effect of scenario was found, *F* (2.34,67.86) = 39.39, *p *< .001, Greenhouse–Geisser corrected. Post-hoc pairwise comparisons indicate that targets were rated less favorably in the compliment and waiting scenarios compared with all other scenarios, *p *< .05, but there was no difference between the story and joke scenarios, *p* = .79. For the non-autistic group, a main effect of scenario was found, *F*(3,87) = 65.90, *p *< .001. Post-hoc pairwise comparisons showed that targets in the waiting scenario were rated as less socially favorable than those in the other three scenarios, *p *< .001.

**Fig. 7:**
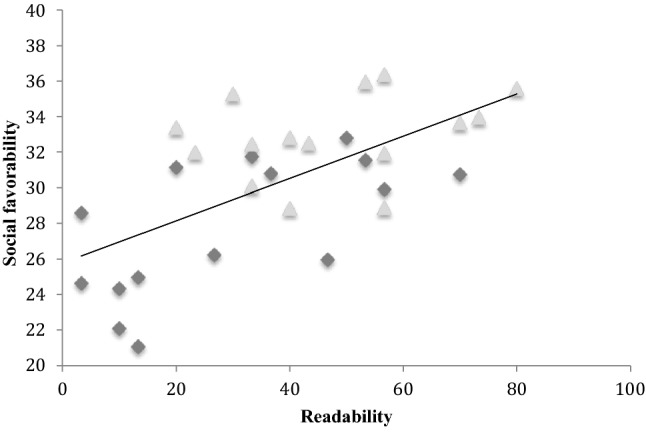
The correlation between readability and social favorability across autistic and non-autistic targets (waiting scenario excluded). The diamonds represent autistic targets and triangles represent non-autistic targets

A bivariate correlation was conducted between target readability (the same data from Sheppard et al. 2016, also presented in Study 1) and social favorability as rated by perceivers in Study 2 (see Fig. 7). We again excluded the waiting scenario, for the same reasons identified in Study 1. In replication of Study 1, based on 30 pairs of values, a positive relationship emerged between target social favorability and target readability, *r* = 0.63, *p *< .001. A partial correlation controlling for target group (autistic or non-autistic) confirmed that the relationship between social favorability and readability survived, *r *= 0.52, *p *= .004. Hence, it seems the correlation is not driven by autistic targets being unreadable and being perceived unfavorably, while typical targets are readable and also perceived favorably. As with Study 1, the significant relationship transcends clinical status.

## General Discussion

The results replicate and extend previous research. Notably, the data offer the first empirical demonstration of a link between autistic individuals being difficult to read and being perceived unfavorably by non-autistic others. After reconfiguring data from Sheppard et al. (2016) we confirmed that when the target was the unit of analysis instead of the perceiver, group differences in readability for autistic and non-autistic targets were still apparent. The results also replicate and extend Sasson et al. (2017), demonstrating that non-autistic perceivers rate non-autistic targets more socially favorably than they rate autistic targets. The rating questions were modified slightly for the current research and the samples of target behavior were rather different compared with Sasson et al., including reactions to multiple different situations. The current findings thus extend what Sasson et al. reported by demonstrating robustness in the face of changes to questions posed to targets and the particulars of the sample of target behavior.

Nevertheless, perceivers were influenced by the situation targets experienced in how they rated target favorability, which suggests that how the target behaves (which is affected by the situation) and not just how they look, influences perceiver ratings. This was most notable in the waiting scenario where targets appeared disagreeable because the experimenter was behaving rudely and perceivers attributed disagreeable target dispositions on witnessing negative target signals. Even when the scenario was disclosed, perceivers persisted in rating targets negatively in this scenario.

When information about the target’s situation was concealed, non-autistic and autistic targets were perceived equally negatively in the waiting scenario. This is a striking finding given that autistic people have been judged more negatively across various other circumstances, including the other scenarios within this study and in previous research (Sasson et al. 2017; Grossman et al. 2013), and demonstrates that there may be circumstances where autistic people are not perceived more negatively than non-autistic people. However, when the situation was disclosed to perceivers, non-autistic targets, whilst still rated rather negatively, were nevertheless rated more positively than autistic targets. It seems situational information had something of a discounting effect in respect of perceived favorability of the behavior of non-autistic targets but not of autistic targets. Perhaps perceivers view more favorably those individuals who respond in a way they expect in a given situation. If non-autistic targets make more typical reactions to these scenarios (see Sheppard et al. 2016), then this may confer increased favorability, even where the response is a negative one.

Having assigned a readability and global social favorability score to each target, it was then possible to perform a correlation analysis to test the association between these two variables. The results confirmed the presence of such an association, a finding that was replicated across two studies, where the scenario targets were experiencing was concealed from perceivers (Study 1) and where it was disclosed (Study 2). This suggests it made no difference to perceivers’ tendency to rate non-autistic targets more favorably than autistic targets whether or not they (perceivers) had some contextual information to assist in explaining target behavior. The association between readability and favorability survived even when clinical diagnosis was partialled out, raising the possibility that the condition of autism is not driving this association.

The statistical association between readability and perceived social favorability does not illuminate the direction of causality or whether the association is the product of a mediating variable. At least, though, we are able to eliminate target diagnostic status as such a mediator. Determining causality is a matter for future research, where larger data sets captured at multiple time points might enable statistical modelling of the directional influence of one variable (e.g. readability) over the other (e.g. favorability).

In anticipation of further research, and to speculate, there are at least two possible explanations which suppose that being unreadable causes one to be perceived unfavorably, a *reward* explanation and a *trait* explanation. Anders et al. (2016) found that perceivers’ attraction to a specific target directly correlated with their self-reported confidence that they had correctly identified the target’s emotional state. They argue that where an interpersonal encounter is rewarding, the reward will be associated with that particular interactant. In other words, when interacting with an individual we feel we can understand well, this will result in greater reward association with that particular individual, generating greater feelings of interpersonal attraction. In support of this reward interpretation, the researchers found that activation in ventral striatum and mOFC (core areas of the brain’s reward system) predicted individual changes in interpersonal attraction. Following this line of argument, a person who is less readable by the majority of the population would consequently be associated with lower reward values, and would be less liked by perceivers from that population.

The trait explanation posits that ‘readability’ may be directly associated with other personality traits. A person who is difficult to read is, by definition, someone, who is not transparent. Such a person, therefore, might seem unpredictable, untrustworthy and incomprehensible, amongst other things. Given there are strong correlations between judgments made on the wide variety of social favorability scales used in this study, it seems reasonable to suggest that people may extrapolate from specific personality characteristics associated with being hard to read to other less favorable characteristics. As with other components of favorability, in this study autistic targets were perceived less trustworthy than neurotypical targets—a finding which is consistent with the suggestion offered here. However, Sasson et al. (2017) did not find any difference between autistic and neurotypical targets in ratings of trustworthiness, perhaps because a different kind of scenario was involved. Nevertheless, this might lead us to question whether the association between readability and favorability is driven by perceptions of trustworthiness.

Causality may actually work in the opposite direction such that being perceived less socially favorable could result in an individual being less readable. Perhaps when a target is perceived negatively, the perceiver is less motivated to empathise and consider the target’s inner state. This in turn could then result in the target being apparently less readable as perceivers made less effort to interpret or understand that individual’s behavior. This interpretation is consistent with previous research that reports mind reading accuracy improves with motivation (Thomas and Maio 2008). It is also possible that both effects are present: there could be a bi-directional relationship between a target’s readability and social favorability, where each influences the other.

A further possibility is that some other variable mediates the relationship between readability and social favorability. In order for the target’s readability to directly influence how socially favorable they are perceived (either via a reward or trait route), this would require perceivers first to make some kind of judgment about how readable the target they are viewing actually is. This might happen in Study 2, where perceivers were told the situation to which the target is reacting: perceivers may notice a mis-match between a target’s behavior and expected behavior in that scenario and infer that they are unable to read the target. It is less apparent how perceivers could infer readability in Study 1, where no contextual information was provided. Perhaps the behavior of some targets is unusual in *any* scenario. This could lead to other people having difficulty inferring what caused the behavior (in readability studies) and also result in more negative social favorability judgments (in favorability studies). In short, perhaps autistic people behave in a way that is perceived by non-autistic people as being ‘out of the ordinary’, rendering them unreadable and also causing them to be perceived unfavorably. Here, ‘out of the ordinary’ is a mediating variable that explains the correlation between unreadability and perceived unfavorability without needing to posit a direct causal link between the two variables.

The possibility that behavior is perceived as being ‘out of the ordinary’ leads to suggestions for future research into why autistic people are difficult to read; and at least two rival explanations are worth considering. One possibility raised here is that the behavior of autistic people is rather different from that of neurotypical people irrespective of context, as would be the case if autistic people had flattened affect and thus were inexpressive such that the signal in their behavior was weakened. However, we already know this is not true of the targets who served in this study (Sheppard et al. 2016)—at least in some scenarios, autistic targets were just as expressive as neurotypical targets. Still, autistic targets, although expressive, might exhibit stereotyped behavior in any scenario that does not encapsulate a signal that is easy to read. Another possibility is that autistic people emit a variety of signals but ones that are misleading, for example, frowning on hearing a joke or appearing bored on hearing a person relate the story of their difficult day.

In future research, if each target experiences a variety of scenarios, it will be possible to determine if autistic people emit the same stereotyped response to all scenarios. If they do so, this would lend support to the suggestion that autistic people behave in a way that is ‘out of the ordinary’ in social contexts. In this case, we would expect perceiver judgments to be around chance level or at best only slightly above chance level. In contrast, if autistic participants emit misleading signals, then perceivers will make systematic errors, in which case accuracy in inferring the scenario will not be at chance level but will be significantly below chance level.

If being unreadable is associated with being rated socially unfavorable, as suggested by our findings, it could in turn have very negative consequences for the development of autistic individuals. This follows if being perceived unfavorably is a barrier to inclusion in the social world, where autistic people, who are in the population minority, instead are condemned to isolation. The social arena provides the experiences essential to understanding how other people behave, think and feel. In being excluded from such experiences, autistic people are trapped in a ‘vicious circle,’ lagging further and further behind in their developing understanding of the psychology of other people, making it ever more difficult for them to be accepted and included (Mitchell 2017). This undesirable effect could well be a risk to quality of life, especially poor mental health, among autistic people (Cassidy and Rodgers 2017). In short, perhaps it is timely to heed the words of Karmiloff-Smith (1998) that development itself is key to understanding developmental disorders, a view amplified by López (2015) specifically in relation to understanding how autism changes over the lifespan, connected with the critical role of social context in these changes.
